# Aerobic Exercise Training Response in Preterm-Born Young Adults with Elevated Blood Pressure and Stage 1 Hypertension: A Randomized Clinical Trial

**DOI:** 10.1164/rccm.202205-0858OC

**Published:** 2022-12-02

**Authors:** Holger Burchert, Winok Lapidaire, Wilby Williamson, Annabelle McCourt, Cameron Dockerill, William Woodward, Cheryl M. J. Tan, Mariane Bertagnolli, Afifah Mohamed, Maryam Alsharqi, Henner Hanssen, Odaro J. Huckstep, Paul Leeson, Adam J. Lewandowski

**Affiliations:** ^1^ Oxford Cardiovascular Clinical Research Facility, Division of Cardiovascular Medicine, Radcliffe Department of Medicine, John Radcliffe Hospital, University of Oxford, Oxford, United Kingdom;; ^2^ School of Physical and Occupational Therapy, McGill University, Montréal, Quebec, Canada;; ^3^ Faculty of Health Sciences, The National University of Malaysia, Kuala Lumpur, Malaysia;; ^4^ Department of Sport, Exercise and Health, University of Basel, Basel, Switzerland; and; ^5^ Department of Biology, U.S. Air Force Academy, Colorado Springs, Colorado, United States

**Keywords:** preterm birth, cardiopulmonary, randomized clinical trial, exercise intervention, aerobic training

## Abstract

**Rationale:**

Premature birth is an independent predictor of long-term cardiovascular risk. Individuals affected are reported to have a lower rate of $\dot{\text{V}}$o_2_ at peak exercise intensity ($\dot{\text{V}}$o_2PEAK_) and at the ventilatory anaerobic threshold ($\dot{\text{V}}$o_2VAT_), but little is known about their response to exercise training.

**Objectives:**

The primary objective was to determine whether the $\dot{\text{V}}$o_2PEAK_ response to exercise training differed between preterm-born and term-born individuals; the secondary objective was to quantify group differences in $\dot{\text{V}}$o_2VAT_ response.

**Methods:**

Fifty-two preterm-born and 151 term-born participants were randomly assigned (1:1) to 16 weeks of aerobic exercise training (*n* = 102) or a control group (*n* = 101). Cardiopulmonary exercise tests were conducted before and after the intervention to measure $\dot{\text{V}}$o_2PEAK_ and the $\dot{\text{V}}$o_2VAT_. A prespecified subgroup analysis was conducted by fitting an interaction term for preterm and term birth histories and exercise group allocation.

**Measurements and Main Results:**

For term-born participants, $\dot{\text{V}}$o_2PEAK_ increased by 3.1 ml/kg/min (95% confidence interval [CI], 1.7 to 4.4), and the $\dot{\text{V}}$o_2VAT_ increased by 2.3 ml/kg/min (95% CI, 0.7 to 3.8) in the intervention group versus controls. For preterm-born participants, $\dot{\text{V}}$o_2PEAK_ increased by 1.8 ml/kg/min (95% CI, −0.4 to 3.9), and the $\dot{\text{V}}$o_2VAT_ increased by 4.6 ml/kg/min (95% CI, 2.1 to 7.0) in the intervention group versus controls. No significant interaction was observed with birth history for $\dot{\text{V}}$o_2PEAK_ (*P* = 0.32) or the $\dot{\text{V}}$o_2VAT_ (*P* = 0.12).

**Conclusions:**

The training intervention led to significant improvements in $\dot{\text{V}}$o_2PEAK_ and $\dot{\text{V}}$o_2VAT_, with no evidence of a statistically different response based on birth history.

Clinical trial registered with www.clinicaltrials.gov (NCT02723552).

**At a Glance Commentary**

**Scientific Knowledge on the Subject**

Preterm birth is associated with long-term cardiopulmonary risk. Young adults born preterm are reported to have a lower rate of $\dot{\text{V}}$o_2_ at peak exercise intensity and at the ventilatory anaerobic threshold compared with their term-born peers, but little is known about their response to exercise training.

**What This Study Adds to the Field**

In this trial subgroup analysis of young adults with elevated blood pressure and stage 1 hypertension, a 16-week aerobic exercise training intervention led to significant improvements in peak exercise $\dot{\text{V}}$o_2_ and ventilatory anaerobic threshold, with no evidence of a statistically different response based on preterm birth history.

Preterm birth affects 10% of live births worldwide (1), with survival rates into adulthood continuing to improve (2). Being born preterm has now been identified as an independent risk factor for cardiovascular (3–6) and pulmonary vascular diseases (7, 8). In line with this, young adults who were born preterm have been shown to have early signs of cardiopulmonary remodeling (9), including limitations under physiological stress conditions (8).

Whole-body $\dot{\text{V}}$o_2_ measured at the ventilatory anaerobic threshold ($\dot{\text{V}}$o_2VAT_) and at peak exercise intensity ($\dot{\text{V}}$o_2PEAK_) are markers of exercise capacity and surrogate measures of cardiovascular-related morbidity and mortality (10, 11). Preterm-born individuals have a lower $\dot{\text{V}}$o_2PEAK_ (12), whereas the results for $\dot{\text{V}}$o_2VAT_ remain inconclusive (13–19). Given that $\dot{\text{V}}$o_2_ is the product of heart rate, stroke volume, and the arterio-mixed venous oxygen difference (20), respiratory and cardiovascular impairments are likely to affect multiple, interrelated components of the oxygen uptake, transport, and utilization chain (21). Indeed, evidence of an impaired cardiac response in preterm-born individuals during exercise is accumulating (8, 15, 22, 23), and mechanical ventilatory constraints have been shown to partly explain reductions in exercise endurance in this population group (24). For instance, Yang and colleagues recently reported that preterm-born individuals have a reduced $\dot{\text{V}}$o_2PEAK_, likely resulting from the combined effects of impaired lung function and altered heart structure (25)_._

Cardiovascular fitness in early life has been shown to be important for long-term health. Shah and colleagues found that each 1-minute reduction in exercise test duration from baseline to year 7 of follow-up was associated with a 21% increased risk in all-cause mortality and a 20% increased risk of cardiovascular disease in long-term follow-up (26). To the best of our knowledge, only one exercise intervention study has been conducted with people who were born preterm, which was conducted in early childhood and did not use the gold standard measure of cardiopulmonary exercise testing to assess exercise capacity (27). Therefore, we performed a prespecified exploratory subgroup analysis of preterm-born young adults with elevated blood pressure in the Trial of Exercise to Prevent HypeRtension in young Adults (TEPHRA) (28, 29). The primary aim of TEPHRA was to reduce blood pressure in young adults with a 16-week aerobic exercise training intervention. However, no effect on systolic or diastolic blood pressure was found, although the $\dot{\text{V}}$o_2PEAK_ was significantly increased (29). The purpose of this subgroup analysis was to explore whether the effect of the exercise intervention on $\dot{\text{V}}$o_2PEAK_ and $\dot{\text{V}}$o_2VAT_ differed between young adults born at term and those born preterm. On the basis of previous evidence of cardiopulmonary limitations during exercise (22, 23), we hypothesized that those who were born preterm would have impaired adaptability in $\dot{\text{V}}$o_2PEAK_ and $\dot{\text{V}}$o_2VAT_ in response to the exercise training intervention compared with term-born young adults.

## Methods

### Study Design and Participants

**Figure 1.**
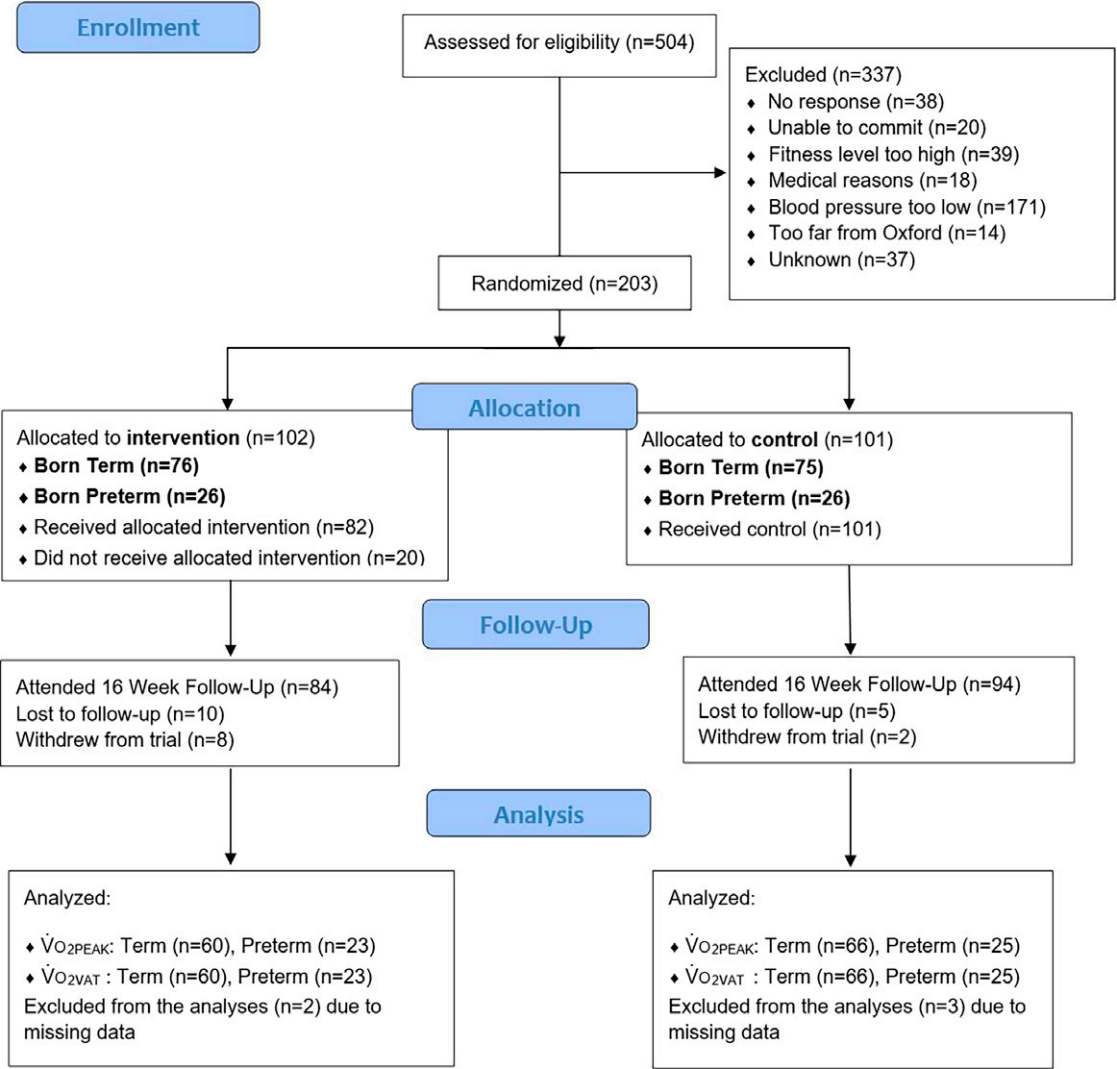
Study flow diagram. $\dot{\text{V}}$o_2PEAK_ = $\dot{\text{V}}$o_2_ measured at peak exercise intensity; $\dot{\text{V}}$o_2VAT_ = $\dot{\text{V}}$o_2_ measured at the ventilatory anaerobic threshold.

TEPHRA is a single-center, open-label, parallel-arm, randomized clinical trial including young adults. Fifty-two preterm- and 151 term-born participants were randomly assigned with minimization according to sex, age (<24 yr, 24–29 yr, and 30–35 yr), and gestational age at birth (<32 wk, 32–37 wk, and >37 wk), in a 1:1 ratio to a control group or an aerobic training and physical activity intervention. The recruitment strategy was aimed to recruit a higher percentage of preterm-born participants than in the general population. The exercise intervention comprised a 16-week aerobic training program, self-monitoring with a wrist-worn physical activity device, and motivational coaching. The control group was offered lifestyle educational materials. A flow diagram of participants’ enrollment and allocation is presented in Figure 1.

The main objective of TEPHRA was to compare the effect of a 16-week supervised aerobic exercise training intervention with usual care/minimal intervention on ambulatory blood pressure levels in young adults with high-normal or elevated blood pressure (28, 29). The primary outcome variable was a 24-hour awake ambulatory blood pressure change (systolic or diastolic) from baseline to 16 weeks (28). Secondary objectives consisted of the investigation of associations between baseline cardiovascular phenotypes, including the preterm-born phenotype, and response to exercise intervention across outcomes (28). Related measures were oxygen uptake kinetics across submaximal and peak exercise at baseline and 16-week follow-up, as described in Table 1 of the published study protocol (28). Full details of the study’s (secondary) objectives, outcome measures, and procedures can be found in the study protocol. No changes were made to the study outcomes after publication of the study protocol.

**Table 1.** Baseline Cohort Characteristics

Characteristic	Preterm			Term		
	Intervention (*n* = 26)	Control (*n* = 26)	*P* Value	Intervention (*n* = 76)	Control (*n* = 75)	*P* Value
Gestational age						
Average gestational age, wk, median (IQR)	32 (30–34)	32 (30–35)	—	40 (39–40)	40 (1.4)	—
⩾37 wk, *n* (%)	0 (0)	0 (0)	—	75 (98.7)	74 (98.7)	—
32–37 wk, *n* (%)	16 (61.5)	16 (61.5)	—	0 (0)	0 (0)	—
<32 wk, *n* (%)	10 (38.5)	10 (38.5)	—	0 (0)	0 (0)	—
Anthropometrics, median (IQR)						
Age, yr	29 (25–33)	28 (25–33)	0.89	28 (25–30)	27 (24–31)	0.89
Height, cm	173 (166–181)	169 (159–176)	0.12	173 (167–178)	171 (167–179)	0.94
Weight, kg	74 (64–80)	68 (59–84)	0.44	73 (13.8–82)	74 (65–83)	0.73
BMI, kg/m^2^	23 (22–26)	23 (21–28)	0.93	25 (22–28)	25 (23–27)	0.80
Demographics						
Male, *n* (%)	13 (50)	12 (46.2)	>0.99	37 (49.3)	35 (47.3)	>0.99
Unemployed, *n* (%)	0 (0)	1 (3.8)	>0.99	1 (1.3)	1 (1.4)	>0.99
Family history of CVD, *n* (%)	15 (57.7)	11 (42.3)	0.28	32 (42.7)	30 (40.5)	0.67
University degree, *n* (%)	14 (53.8)	18 (69.2)	0.36	61 (81.3)	60 (81.1)	0.85
Smoker, *n* (%)	3 (11.5)	4 (15.4)	>0.99	7 (9.3)	7 (9.5)	0.76
Units of alcohol per wk, median (IQR)	2.5 (0.0–8.0)	4.0 (1.5–8.7)	0.21	4.0 (0.0–8.8)	3.2 (0.0–8.2)	0.78
Biochemistry and blood pressure						
Cholesterol HDL ratio, median (IQR)	3.3 (2.7–3.9)	3.2 (2.3–4.5)	0.49	3.3 (2.7–4.0)	3.2 (2.8–4.0)	0.83
Glucose (mmol), median (IQR)	4.8 (4.6–5.1)	4.9 (4.3–5.1)	0.87	4.7 (4.5–5.1)	4.8 (4.5–5.3)	0.17
HOMA insulin resistance, median (range)	0.8 (0.6–1.1)	0.8 (0.5–1.1)	0.93	1.0 (0.7–1.3)	0.8 (0.6–1.3)	0.18
24 h SBP (mm Hg)	129 (10)	128 (9)	—	129 (9)	128 (9)	0.56
24 h DBP (mm Hg)	77 (7)	77 (8)	0.71	77 (6)	78 (7)	0.64
Lung function/exercise						
FVC, L, median (IQR)	4.7 (4.0–5.8)	4.5 (3.5–5.8)	0.23	4.4 (4.0–5.3)	4.7 (3.9–5.4)	0.32
FVC, *z*	0.1 (1.1)	−0.2 (0.9)	0.24	−0.2 (0.9)	0.1 (1.2)	**0.05**
FVC % predicted	102 (14)	98 (11)	0.31	96 (16)	102 (15)	**0.02**
FEV_1_, L, median (IQR)	3.8 (3.3–4.7)	3.5 (2.8–4.1)	0.25	3.8 (3.3–4.3)	3.9 (3.3–4.6)	0.20
FEV_1_, *z*	−0.2 (1.0)	−0.5 (1.2)	0.30	−0.4 (0.9)	0.1 (1.2)	**0.02**
FEV_1_ % predicted	97 (13)	93 (15)	0.31	94 (16)	101 (15)	**0.001**
FEV_1_/FVC, median (IQR)	0.8 (0.7–0.9)	0.8 (0.8–0.9)	0.96	0.8 (0.8–0.9)	0.9 (0.8–0.9)	0.69
FEV_1_/FVC *z*	−0.5 (1.2)	−0.5 (1.2)	0.94	−0.2 (0.9)	−0.1 (0.9)	0.55
FEV_1_/FVC % predicted	95 (88–103)	97 (90–103)	0.92	100 (94–103)	100 (94–104)	0.57
Peak $\dot{\text{V}}$o_2_, L, median (IQR)	2.2 (2.0–2.8)	2.4 (1.8–2.6)	0.67	2.4 (2.0–2.9)	2.6 (2.1–3.0)	0.12
Peak $\dot{\text{V}}$o_2_, ml/kg/min, median (IQR)	34 (28–39)	33 (28–36)	0.88	32 (27–38)	35 (31–40)	**0.05**
Peak $\dot{\text{V}}$o_2_ % predicted	83 (14)	81 (15)	0.75	81 (14)	86 (16)	0.10
Peak HR, median (IQR)	181 (179–187)	179 (174–188)	0.33	181 (173–189)	180 (171–189)	0.58
Peak RER*	1.2 (0.04)	1.1 (0.05)	0.61	1.1 (0.05)	1.1 (0.06)	0.09
$\dot{\text{V}}$o_2VAT_, L/min	1.4 (0.5)	1.3 (0.6)	0.61	1.4 (0.5)	1.6 (0.6)	**0.02**
$\dot{\text{V}}$o_2VAT_, ml/kg/min	19 (6)	19 (5)	0.74	18 (6)	21 (7)	**0.008**
HR_VAT_, bpm	129 (17)	126 (18)	0.51	128 (19)	136 (22)	**0.02**
SVI_VAT.est_, ml/m^2^	49 (9)	50 (13)	0.77	48 (10)	50 (10)	0.19
SVI_PEAK_, ml/m^2^	45 (10)	45 (10)	0.95	44 (9)	48 (10)	**0.03**
CI_VAT_, ml/min/m^2^	6,330 (1,590)	6,180 (1,530)	0.73	6,180 (1,500)	6,870 (1,730)	**0.01**
CI_PEAK_, ml/min/m^2^	8,160 (1,760)	8,060 (1,850)	0.84	7,970 (1,670)	8,550 (1,820)	**0.04**
VE_VAT_, L/min	38 (14)	36 (14)	0.57	38 (14)	42 (17)	0.13
BF_VAT_, breaths/min	22 (6)	24 (6)	0.35	24 (6)	24 (6)	0.98
VT_VAT_, L	1.8 (0.6)	1.5 (0.5)	0.14	1.6 (0.5)	1.8 (0.6)	0.07

*Definition of abbreviations*: BF_VAT_ = breathing frequency at the ventilatory anaerobic threshold; BMI = body mass index; bpm = beats per minute; CI_PEAK_ = cardiac index at peak exercise intensity; CI_VAT_ = cardiac index at the ventilatory anaerobic threshold; CVD = cardiovascular disease; DBP = diastolic blood pressure; HDL = high-density lipoprotein; HOMA = Homeostatic Model Assessment; HR = heart rate; IQR = interquartile range; RER* = respiratory exchange ratio; SBP = systolic blood pressure; SVI_PEAK_ = stroke volume index at peak exercise intensity; SVI_VAT.est_ = stroke volume index at the ventilatory anaerobic threshold; VE_VAT_ = minute ventilation at the ventilatory anaerobic threshold; $\dot{\text{V}}$o_2VAT_ = $\dot{\text{V}}$_O_2__ measured at the ventilatory anaerobic threshold; VT_VAT_ = tidal volume at the ventilatory anaerobic threshold; *z* = *z*-score.

Data are presented as mean (SD). Frequencies are presented as *n* (%). RERs* are presented as mean (SD). Bold *P* values are statistically significant (*P* < 0.05).

Participants were recruited through open recruitment; General Practitioner records; invitations from hospital birth registers; online advertising on Facebook, Instagram, and Twitter; and invitations after participation in previous studies. Eligibility criteria comprised the following: participants were 18 to 35 years old; had a 24-hour awake ambulatory systolic and/or diastolic blood pressure higher than 115/75 mm Hg and lower than 159/99 mm Hg; had a body mass index less than 35 kg/m^2^; were not on and had not previously been prescribed hypertension medications; had verifiable birth history of preterm birth (<37 wk) or full-term birth (⩾37 wk); and had the ability to access and use a computer and the Internet. Gestational age and birth weight were verified by medical records or personal child health records. Exclusion criteria comprised the following: being pregnant; having participated in structured exercise more than once per week or maintained high cardiovascular fitness; any contraindication to exercise; the inability to walk briskly on the flat for 15 minutes; and any evidence of cardiomyopathy, inherited cardiac abnormalities, or other significant cardiovascular disease. The study was conducted in the University of Oxford’s Division of Cardiovascular Medicine sites at the John Radcliffe Hospital in Oxford, UK. Enrollment occurred between June 30, 2016 and October 26, 2018; the final follow-up was completed on January 9, 2020.

The trial protocol and any subsequent amendments were approved by the University of Oxford as host institution and study sponsor and by the South Central Research Ethics Committee for the National Health Service Health Research Authority (Reference no. 16-SC-0016). A trial steering committee and independent data and safety monitoring board monitored the study, and all participants provided written informed consent. The investigators ensured that the study was conducted in accordance with the principles of the Declaration of Helsinki as well as in accordance with relevant regulations and good clinical practice.

### Anthropometry

Height and weight were measured to the nearest centimeter and 0.1 kg, respectively, with participants’ footwear removed and light clothing worn. With a measuring tape, waist circumference was measured 2 cm above the iliac crest, and hip circumference was measured at the point of widest overall girth near the level of the greater trochanter of the femur or midbuttock.

### Spirometry and Cardiopulmonary Exercise Test

All participants underwent spirometry testing according to standard guidelines by using the Cortex Metalyzer 3B (Cortex Biophysik GmbH) (30). Before each test, the spirometer underwent calibration checks of the flow and volume with a 3.0-L calibration syringe (Futuremed). Each participant was seated and guided through the test while wearing a nose clip, with their lips tightly sealed around the mouthpiece. At least three acceptable forced expiratory maneuvers were conducted with every participant. Visual checks of the waveforms were performed to ensure consistency and data quality before the highest values were selected and recorded on a clinical research form. Parameters recorded included FEV_1_ and FVC (23). FEV_1_ and FVC *z*-scores and percent predicted values were calculated using the Global Lung Initiative online calculator (http://gli-calculator.ersnet.org/).

Afterward, participants completed a peak cardiopulmonary exercise test on a seated stationary cycle ergometer (Ergoline GmbH) using a validated incremental protocol. Respiratory gases were measured breath by breath using the same Metalyzer as that used for spirometry. Continuous electrocardiogram monitoring was used to record heart rate, and blood pressure readings were taken every 4 minutes with a manual mercury sphygmomanometer (ACCOSON Freestyle). Participants maintained 60 revolutions per minute during the test, which started with one quiescent minute of resting measurements followed by 2 minutes of warm-up at 20 watts. After this, workload increased to 35 watts. To normalize test duration to 8–12 minutes, participants who reported higher activity or fitness levels had their workload increased to 75 watts after the warm-up. The workload was then increased in increments of 15 watts each minute, while participants cycled continuously until exhaustion prevented them from maintaining 50 revolutions per minute or safety termination criteria were met. The test ended with a 2-minute cool-down period at 50 watts and revolutions of the participant’s own preference.

### Intervention

The intervention stipulated three 60-minute aerobic training sessions (completed on bicycle ergometers) on separate days per week for 16 weeks at an exercise intensity of 60–80% of peak heart rate measured at baseline. A wrist-worn heart rate and activity monitor (Fitbit Charge HR; Fitbit, Inc.) was gifted to the participants who were encouraged to wear it daily. To track physical activity in the intervention group, activity from the wrist-worn activity monitor was tracked using the Fitabase data management platform and records kept of training sessions attended. The compliance threshold for the intervention was set at 80%, equivalent to 39 or more independent training sessions, with no more than 2 weeks between sessions. A compliant session was defined, *a priori*, as a supervised one. This definition was refined by the trial committees during the trial to being an aerobic session defined as a supervised gym session 40 to 60 minutes long; a self-reported aerobic session 40 to 60 minutes long; a day with a step count of 8,000 steps or more measured by their Fitbit; or a total of 40 or more Fitbit active minutes that were defined as fairly and vigorously active. After the 16 weeks of intervention, participants attended a 60-minute motivational coaching session where they reflected on their physical activity behavior as well as the intervention to set long-term physical activity goals. The intervention team was trained in motivational coaching and consisted of physiologists, physiotherapists, clinical nurse specialists, and a physician.

### Statistical Analysis

Baseline subgroup cohort characteristics were tested for normality by visual inspection of histograms and normal quantile-quantile plots. Continuous variables are presented as mean (standard deviation) when the data were normally distributed and median (interquartile range) when the data were non-normally distributed. Frequencies are presented as *n*s with percentages. Wilcoxon rank-sum tests were applied on the continuous data and chi-square tests for the frequencies. All analyses were conducted in R, version 3.6.1 (July 5, 2019).

The main effects of the trial were analyzed by fitting general linear models with 16-week follow-up $\dot{\text{V}}$o_2PEAK_ and $\dot{\text{V}}$o_2VAT_ values as outcome measures, adjusting for baseline values of the outcome measure and minimization factors (age as continuous variable; sex; and gestation ⩾37, or <37 weeks’ gestation). For instance, the regression equation for adjusted $\dot{\text{V}}$o_2PEAK_ was as follows:

    (1) $$\begin{array}{l} 16\;\text{Weeks}\;{\dot{V}}_{O_{2P\text{EAK}}}\;=\;\beta_{0}\;+\;\beta_{1}B\text{aseline}\;{\dot{V}}_{O_{2P\text{EAK}}}\;\\ \;\;+\;\beta_{2}Sex\;+\;\beta_{3}Age\;+\;\beta_{4}G\text{estation}\;\\ \;\;+\;\beta_{5}A\text{llocation}. \end{array}$$

Subgroups (term- and preterm-born) were defined by splitting the gestational age variable from the minimization procedure (gestation: <32, 32–37, or ⩾37 weeks’ gestation) into two levels: ⩾37 and <37 weeks’ gestation. The subgroup analysis was then performed by fitting additional linear models, including an interaction term between treatment allocation (exercise, control) and gestational age category (⩾37 or <37 weeks’ gestation) to test whether the intervention effects would vary significantly across subgroups. Hence, for adjusted $\dot{V}$o_2PEAK_, the regression equation was as follows:

    (2) $$\begin{array}{l} 16\;\text{Weeks}\;{\dot{V}}_{O_{2P\text{EAK}}}\;=\;\beta_{0}\;+\;\beta_{1}B\text{aseline}\;{\dot{\text{V}}}_{O_{2P\text{EAK}}}\\ \;\;+\;\beta_{2}Sex\;+\;\beta_{3}Age\;+\;\beta_{4}G\text{estation}\\ \;\times \;\beta_{5}A\text{llocation}. \end{array}$$

Complete case analyses were performed throughout. Model assumptions were tested and confirmed by plotting residuals versus fitted values. The results for preterm and term groups are presented as adjusted mean differences with 95% confidence intervals (CI). An α level of 0.05 was considered statistically significant. No adjustments were done for multiple testing. The cardiac index and stroke volume index (SVI) at the ventilatory anaerobic threshold and peak exercise intensity (CI_VAT_, SVI_VAT_, CI_PEAK_, and SVI_PEAK_, respectively) were estimated according to Stringer, Hansen, and Wasserman on the basis of oxygen uptake (31) and then indexed to body surface area according to the Du Bois formula.

## Results

### Exercise Intervention: Main Effects

Of the participants who discontinued the study, nine were in the exercise intervention group and five were in the control group. There was no evidence of exercise intolerance being the reason for withdrawal. Cohort characteristics, including gestational age, anthropometrics, demographics, biochemistry, blood pressure, and lung function, as well as peak heart rate and peak respiratory exchange ratio, are shown in Table 1. The cohort was >90% White. Regarding the main effects, adjusted $\dot{\text{V}}$o_2PEAK_ and $\dot{\text{V}}$o_2VAT_ increased significantly after 16 weeks of exercise training by 2.7 ml/kg/min (95% CI, 1.6 to 3.8) and 2.9 ml/kg/min (95% CI, 1.6 to 4.3) in the intervention group compared with controls as calculated by Equation 1. The full regression models are itemized in Table 2.

**Table 2.** Main Effect Models of the Prespecified Subgroup Analyses Comparing the Effect of the 16-Week Aerobic Training Intervention (Intervention vs. Control) on Peak Exercise $\dot{\text{V}}$o_2_ and Ventilatory Anaerobic Threshold

Model	Estimate (95% CI)	*P* Value
Primary outcome		
Peak $\dot{\text{V}}$o_2PEAK_, ml/kg/min, adjusted *R*^2^ = 0.7, *df* = 168		
Baseline $\dot{\text{V}}$o_2PEAK_, ml/kg/min	0.8 (0.7–0.9)	<0.001
Allocation, intervention	2.7 (1.6–3.8)	<0.001
Secondary outcome		
$\dot{\text{V}}$o_2VAT_, ml/kg/min, adj. *R*^2^ = 0.5, *df* = 169		
Baseline $\dot{\text{V}}$o_2VAT_, ml/kg/min	0.7 (0.6–0.8)	<0.001
Allocation, intervention	2.9 (1.6–4.3)	<0.001

*Definition of abbreviations*: 95% CI = 95% confidence interval; $\dot{\text{V}}$o_2PEAK_ = $\dot{\text{V}}$o_2_ measured at peak exercise intensity; $\dot{\text{V}}$o_2VAT_ = $\dot{\text{V}}$_O_2__ measured at the ventilatory anaerobic threshold.

### Exercise Intervention: Preterm- and Term-born Subgroup Interaction Effects

**Figure 2.**
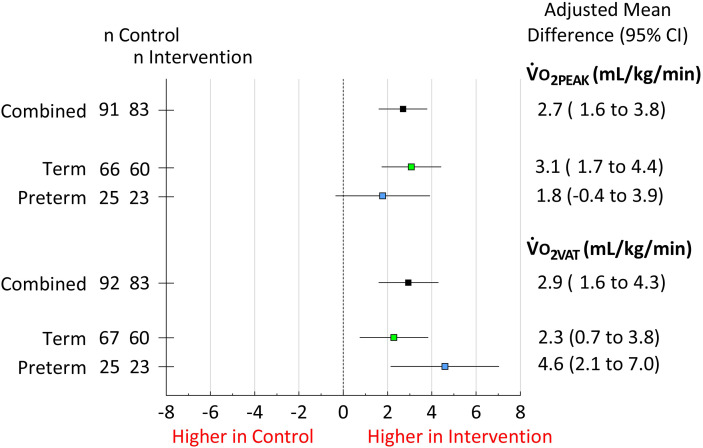
Forest plot showing the main effects of a 16-week aerobic training intervention in the full-study group (black), followed by the subgroup effects for term-born (green) and preterm-born (blue) young adults on the primary outcome ($\dot{\text{V}}$o_2PEAK_) and secondary outcome ($\dot{\text{V}}$o_2VAT_). Subgroup effects did not differ significantly on the basis of interaction analyses (*P* = 0.32 and *P* = 0.12, respectively). 95% CI = 95% confidence interval; $\dot{\text{V}}$o_2PEAK_ = $\dot{\text{V}}$o_2_ measured at peak exercise intensity; $\dot{\text{V}}$o_2VAT_ = $\dot{\text{V}}$o_2_ measured at the ventilatory anaerobic threshold.

Adding an interaction term to the model, as per Equation 2, returned the following subgroup results. In term-born individuals in the intervention group, adjusted $\dot{\text{V}}$o_2PEAK_ increased significantly by 3.1 ml/kg/min (95% CI, 1.7 to 4.4), compared with term-born controls, whereas there was no change in preterm-born participants (interaction effect *P* = 0.32; Figure 2). The adjusted $\dot{\text{V}}$o_2VAT_ significantly increased in the intervention group compared with that in the control group for both the term-born participants (2.3 ml/kg/min; 95% CI, 0.7 to 3.8) and the preterm-born participants (4.6 ml/kg/min; 95% CI, 2.1 to 7.0; interaction effect *P* = 0.12; Figure 2). There was a greater magnitude of increase in $\dot{\text{V}}$o_2VAT_ compared with that of $\dot{\text{V}}$o_2PEAK_ in preterm-born participants, which was not the case for the term-born participants (Figure 2).

### *Post Hoc* Subgroup Analysis

**Figure 3.**
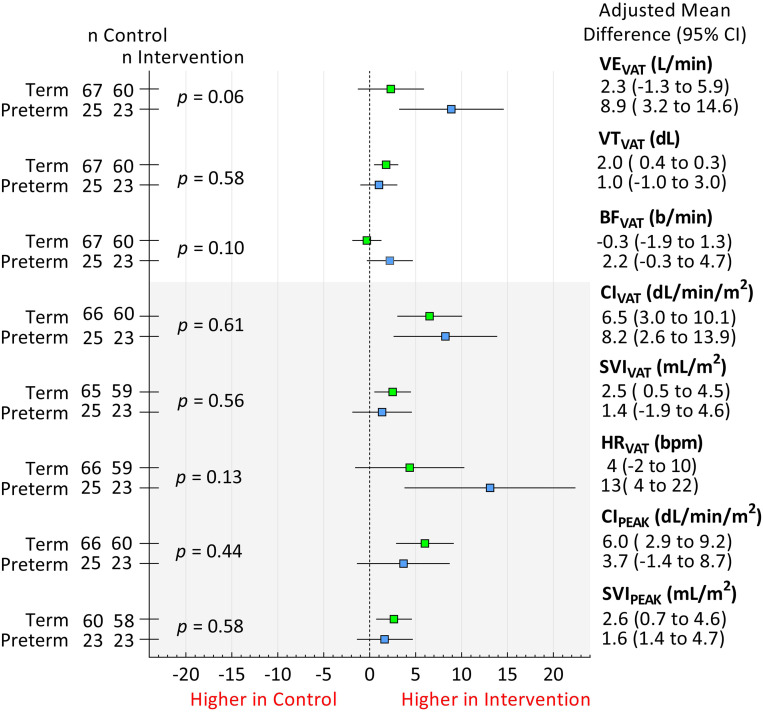
Forest plot of the subgroup effect of a 16-week aerobic training intervention for the *post hoc* analyses comparing term-born (green) and preterm-born (blue) young adults. *P* values represent the interaction analyses for each of the subgroup analyses. BF_VAT_ = breathing frequency at the ventilatory anaerobic threshold; CI_PEAK_ = cardiac index at peak exercise intensity; CI_VAT_ = cardiac index at the ventilatory anaerobic threshold; HR_VAT_ = heart rate at the ventilatory anaerobic threshold; SVI_PEAK_ = stroke volume index at peak exercise intensity; SVI_VAT_ = stroke volume index at the ventilatory anaerobic threshold; VE_VAT_ = minute ventilation at the ventilatory anaerobic threshold; VT_VAT_ = tidal volume at the ventilatory anaerobic threshold.

We conducted eight *post hoc* analyses to further explore cardiopulmonary adaptations. The first four were to test whether, at the ventilatory anaerobic threshold, heart rate (HR_VAT_), minute ventilation (VE_VAT_), breathing frequency (BF_VAT_), and tidal volume (VT_VAT_) would be exclusively increased in preterm-born participants. The remaining four analyses were conducted to test whether SVI_VAT_, SVI_PEAK_, CI_VAT_, and CI_PEAK_ increased less in the preterm-born intervention group compared with preterm-born controls than in the term-born intervention group compared with term-born controls. The main effects for the combined study groups were analyzed as per Equation 1, with the results presented in Table 3. The subgroup effects were again analyzed by using Equation 2, with the results shown in Figure 3.

**Table 3.** Main Effect Models of the *Post Hoc* Analyses Comparing the Effect of the 16-Week Aerobic Training Intervention (Intervention vs. Control) on Exercise Parameters

Model	Estimate (95% CI)	*P* Value
HR_VAT_, bpm, adj. *R*^2^ = 0.35, *df* = 167		
Baseline HR_VAT_, bpm	0.6 (0.5 to 0.8)	<0.001
Allocation, intervention	6.9 (1.9 to 11.9)	0.008
VE_VAT_, L/min, adj. *R*^2^ = 0.5, *df* = 169		
Baseline VE_VAT_, L/min	0.7 (0.6 to 0.8)	<0.001
Allocation, intervention	4.2 (1.1 to 7.2)	0.008
BF_VAT_, breaths/min, adj. *R*^2^ = 0.4, *df* = 169		
Baseline BF_VAT,_ breaths/min	0.6 (0.5 to 0.7)	<0.001
Allocation, intervention	0.4 (−1.0 to 1.7)	0.58
VT_VAT_, L, adj. *R*^2^ = 0.7, *df* = 169		
Baseline VT_VAT_, L	0.8 (−0.7 to 0.9)	<0.001
Allocation, intervention	0.1 (0.4 to 0.3)	0.01
SVI_est.VAT_, ml/m^2^, adj. *R*^2^ = 0.7, *df* = 166		
Baseline SVI_est.VAT_, ml/m^2^	0.7 (0.6 to 0.8)	<0.001
Allocation, intervention	2.2 (0.5 to 3.9)	0.01
SVI_est.PEAK_, ml/m^2^, adj. *R*^2^ = 0.7, *df* = 158		
Baseline SVI_est.PEAK_, ml/m^2^	0.7 (0.6 to 0.8)	<0.001
Allocation, intervention	2.4 (0.7 to 4.0)	0.005
CI_est.VAT_, ml/min/m^2^, adj. *R*^2^ = 0.6, *df* = 168		
Baseline CI_est.VAT_, ml/min/m^2^	0.7 (0.6 to 0.8)	<0.001
Allocation, intervention	702 (403 to 1,001)	<0.001
CI_est.PEAK_, ml/min/m^2^, adj. *R*^2^ = 0.7, *df* = 168		
Baseline CI_est.PEAK_, ml/min/m^2^	0.7 (0.6 to 0.8)	<0.001
Allocation, intervention	535 (267 to 803)	<0.001

*Definition of abbreviations*: adj. *R*^2^ = adjusted *R*^2^; BF_VAT_ = breathing frequency at the ventilatory anaerobic threshold; bpm = beats per minute; CI_est.PEAK_ = cardiac index estimate at peak exercise intensity; CI_est.VAT_ = cardiac index estimate at the ventilatory anaerobic threshold; HR_VAT_ = heart rate at the ventilatory anaerobic threshold; VE_VAT_ = minute ventilation at the ventilatory anaerobic threshold; VT_VAT_ = tidal volume at the ventilatory anaerobic threshold; SVI_est.PEAK_ = stroke volume index estimate at peak exercise intensity; SVI_est.VAT_ = stroke volume index estimate at the ventilatory anaerobic threshold.

Adjusted HR_VAT_ increased significantly by 13 beats per minute (bpm) (95% CI, 3.8 to 22.4) in the preterm-born intervention group compared with preterm-born controls, with no significant change in term-born participants (interaction effect *P* = 0.13; Figure 3). Adjusted VE_VAT_ increased significantly only in the preterm-born intervention group compared with preterm-born controls by 8.9 L/min (95% CI, 3.2 to 14.6), with no significant change in term-born participants (interaction effect *P* = 0.06; Figure 3). Adjusted BF_VAT_ did not change significantly in the preterm-born or term-born intervention group compared with their respective control groups. VT_VAT_ increased significantly in term-born participants in the intervention group compared with term-born controls (2.0 dL; 95% CI, 0.4 to 0.3) whereas there was no significant change in the preterm-born participants (interaction effect *P* = 0.58; Figure 3).

Adjusted SVI_VAT_ showed no significant change in the preterm-born participants, whereas it increased significantly in the term-born intervention group by 2.5 ml/m^2^ (95% CI, 0.5 to 4.5) compared with term-born controls (interaction effect *P* = 0.56; Figure 3). Similarly, adjusted SVI_PEAK_ showed no significant change in the preterm-born participants, whereas it increased significantly in the term-born intervention group by 2.6 ml/m^2^ (95% CI, 0.7 to 4.6) compared with term-born controls (interaction effect *P* = 0.58; Figure 3). Adjusted CI_VAT_ increased significantly by 8.2 dL/min/m^2^ (95% CI, 2.6 to 13.9) in the preterm-born intervention group compared with preterm-born controls and increased significantly in the term-born intervention group by 6.5 dL/min/m^2^ (95% CI, 3.0 to 10.1) compared with term-born controls (interaction effect *P* = 0.61; Figure 3). Adjusted CI_PEAK_ showed no significant change in the preterm-born participants, whereas it increased significantly in the term-born intervention group by 6.0 dL/min/m^2^ (95% CI, 2.9 to 9.2) compared with term-born controls (interaction effect *P* = 0.44; Figure 3).

## Discussion

This was the first analysis exploring the potential benefits of an aerobic exercise training intervention in preterm-born young adults. There was no statistical evidence of a smaller increase in $\dot{\text{V}}$o_2PEAK_ and $\dot{\text{V}}$o_2VAT_ in preterm-born participants compared with term-born controls in response to the intervention. In addition, the increase in $\dot{\text{V}}$o_2VAT_ in the preterm-born intervention group was twice as large as that in the term-born intervention group compared with their respective control groups. However, this difference was not statistically significant, indicating that there was no difference in cardiopulmonary adaptation between preterm- and term-born participants.

The observed greater increase in $\dot{\text{V}}$o_2VAT_ compared with $\dot{\text{V}}$o_2PEAK_ after the intervention in preterm-born participants meant that $\dot{\text{V}}$o_2VAT_ occurred at a higher percentage of $\dot{\text{V}}$o_2PEAK_. Such higher percentages are paradoxically observed in healthy elite athletes as well as in diseased populations, including those with pulmonary hypertension and heart failure (10, 32, 33). The *post hoc* tests of HR_VAT_, VE_VAT_, BF_VAT_, and VT_VAT_ were performed to test whether the $\dot{\text{V}}$o_2VAT_ indeed occurred at a higher percentage of $\dot{\text{V}}$o_2PEAK_. None of them showed significant interactions, although HR_VAT_, VE_VAT_, and BF_VAT_ were consistently higher in the preterm-born intervention group than in the preterm-born control group, which was not the case among the term-born participants and thus further suggests that $\dot{\text{V}}$o_2VAT_ occurred at a higher percentage of $\dot{\text{V}}$o_2PEAK_.

Changes in $\dot{\text{V}}$o_2_ can be due to both systemic and central physiological changes. Evidence of systemic changes in preterm-born populations includes lower muscular fitness in preterm-born individuals (34). Furthermore, postmortem studies of infants born preterm showed lower activity and content of pyruvate dehydrogenase, respiratory chain complexes III and IV, and citrate synthase (35, 36). Subsequently, in rats, Tetri and colleagues demonstrated sex-specific higher skeletal muscle fatigability, lower muscle mitochondrial oxidative capacity, more mitochondrial damage, and greater glycolytic enzyme expression (37). A recent study using the same animal model demonstrated signs of inflammation in skeletal muscles, which associated with muscle fiber atrophy, fiber type shifting from slow- to fast-twitch fibers, and impaired muscle function (38), which persisted into adulthood.

Other studies have shown central cardiovascular changes in preterm-born individuals. It has been observed that preterm-born individuals have smaller cardiac ventricular volumes (39) that are associated with functional cardiac impairments during exercise, which are predictive of their reduced exercise tolerance (8, 15, 22, 23). In patients with heart failure with preserved ejection fraction, training-induced increases in $\dot{\text{V}}$o_2PEAK_ are mainly due to increased oxygen extraction rather than cardiac adaptations (40). Indeed, Haykowski and colleagues showed that changes in cardiac output after 16 weeks of endurance training only explained 16% of the increase in $\dot{\text{V}}$o_2PEAK_, with no improvements in left ventricular systolic or diastolic function (41). In line with this, meta-analysis evidence in patients with heart failure has shown that the anaerobic threshold improves the most after exercise training (42). Given the known stroke volume impairments (22, 43, 44), it is plausible that $\dot{\text{V}}$o_2PEAK_ in the preterm-born intervention group increased primarily by systemic factors. Indeed, this would explain the predominant increase in $\dot{\text{V}}$o_2VAT_ as compared with a change in $\dot{\text{V}}$o_2PEAK_ after the intervention, as the former depends on skeletal muscle factors and the latter depends primarily on the ability to increase cardiac output. However, the statistical nonsignificant differences in $\dot{\text{V}}$o_2PEAK_ and $\dot{\text{V}}$o_2VAT_ indicate that potential central and systemic differences may be small and not of clinical relevance for the majority of preterm-born individuals.

The nonsignificant 13-bpm increase in HR_VAT_ after the intervention among preterm-born participants was surprising but comports with the notion that $\dot{\text{V}}$o_2VAT_ occurred at a higher percentage of $\dot{\text{V}}$o_2PEAK_. Given that, normally, an increase in the ventilatory anaerobic threshold to a higher workload occurs reciprocally to the hallmark effect of exercise training to reduce heart rate at submaximal workloads, one would expect only minor increases in HR_VAT_, if at all (45, 46). The increased HR_VAT_ could mean that expected improvements in the left ventricular SVI may be smaller than for those born at term, such that preterm-born participants stayed reliant on heart rate to increase cardiac output. This observation may support the findings by Corrado and colleagues, who observed that the preterm heart appears to be heart rate dependent but is sensitive to changes in afterload (47). The authors used four-dimensional flow magnetic resonance imaging to investigate cardiac hemodynamics before and after the administration of sildenafil (afterload reduction) and metoprolol (heart rate reduction) in young adults who were born very to extremely prematurely. They showed that sildenafil resulted in an increased cardiac index, mediated by increases in heart rate and stroke volume, whereas metoprolol reduced the cardiac index by 0.37 L/min/m^2^ and the heart rate by 5 bpm. However, the absence of significant interaction effects for SVI_VAT_ and SVI_PEAK_ does not conclusively support the idea that the preterm participants were substantially reliant on heart rate. Also, Haraldsdottir and colleagues did not observe a higher heart rate during submaximal levels of exercise in preterm-born participants compared with their term-born peers, despite an impaired stroke volume during exercise (15), but this was a relatively small sample size that may have resulted in a lack of statistical power (48). Nevertheless, the higher estimates we observed for CI_VAT_ and HR_VAT_ in preterm-born participants warrant further research to better understand the exercise response in people born preterm (22, 23).

## Limitations

Despite being prespecified, this study was an exploratory subgroup analysis and must therefore be regarded as hypothesis generating. As such, we were not able to explore all birth history factors related to preterm birth that may have impacted the response to the trial intervention. The cardiac index and SVI estimates were made on the basis of $\dot{\text{V}}$o_2_ and are consequently not independent from it. It allowed, however, an interpretation of $\dot{\text{V}}$o_2PEAK_ and $\dot{\text{V}}$o_2VAT_ with regard to cardiac function based on a strict method. Furthermore, subgroup analyses as part of the main trial naturally lack statistical power (49), raising the possibility of false negatives. For example, to detect the 13% difference in $\dot{\text{V}}$o_2PEAK_ between preterm- and term-born groups reported from meta-analyses, a minimum of 47 participants in each group (instead of 25 vs. 23 as presented in this study) would be needed when using a two-sided test at a 5% significance level and 80% power (12, 48). Future, adequately powered trials should investigate the potential for negative responses to exercise training and whether the response is dependent on the degree of prematurity. The recruitment approach may also limit the generalizability of the results, given that participants on antihypertensive medication were excluded from the trial and there was a prespecified, purposeful overrepresentation of preterm-born young adults compared with the general population. Finally, although the exercise training program was chosen to replicate interventions identified by a systematic review of randomized controlled trials aiming to reduce blood pressure (28, 50), different intensities or volumes of training may have had larger effects on oxygen uptake and ventilatory threshold.

## Conclusions

In this exploratory hypothesis-generating subgroup analysis of young adults with elevated blood pressure and stage 1 hypertension, preterm-born individuals showed no significant differences compared with term-born individuals in their cardiopulmonary exercise adaptation after 16 weeks of aerobic exercise training. Future larger studies should investigate whether this is a true effect or perhaps due to small numbers.

## References

[1] Chawanpaiboon S Vogel JP Moller AB Lumbiganon P Petzold M Hogan D *et al.* Global, regional, and national estimates of levels of preterm birth in 2014: a systematic review and modelling analysis *Lancet Glob Health* 2019 7 e37 e46 30389451 10.1016/S2214-109X(18)30451-0 PMC6293055

[2] Burchert H Lewandowski AJ Preterm birth is a novel, independent risk factor for altered cardiac remodeling and early heart failure: is it time for a new cardiomyopathy? *Curr Treat Options Cardiovasc Med* 2019 21 8 30762137 10.1007/s11936-019-0712-9

[3] Crump C Howell EA Stroustrup A McLaughlin MA Sundquist J Sundquist K Association of preterm birth with risk of ischemic heart disease in adulthood *JAMA Pediatr* 2019 173 736 743 31157896 10.1001/jamapediatrics.2019.1327 PMC6547251

[4] Carr H Cnattingius S Granath F Ludvigsson JF Edstedt Bonamy AK Preterm birth and risk of heart failure up to early adulthood *J Am Coll Cardiol* 2017 69 2634 2642 28545637 10.1016/j.jacc.2017.03.572

[5] Crump C Groves A Sundquist J Sundquist K Association of preterm birth with long-term risk of heart failure into adulthood *JAMA Pediatr* 2021 175 689 697 33818601 10.1001/jamapediatrics.2021.0131 PMC8022265

[6] Crump C Preterm birth and mortality in adulthood: a systematic review *J Perinatol* 2020 40 833 843 31767981 10.1038/s41372-019-0563-y PMC7246174

[7] Naumburg E Axelsson I Huber D Söderström L Some neonatal risk factors for adult pulmonary arterial hypertension remain unknown *Acta Paediatr* 2015 104 1104 1108 26346500 10.1111/apa.13205

[8] Goss KN Beshish AG Barton GP Haraldsdottir K Levin TS Tetri LH *et al.* Early pulmonary vascular disease in young adults born preterm *Am J Respir Crit Care Med* 2018 198 1549 1558 29944842 10.1164/rccm.201710-2016OC PMC6298636

[9] Mohamed A Lamata P Williamson W Alsharqi M Tan CMJ Burchert H *et al.* Multimodality imaging demonstrates reduced right-ventricular function independent of pulmonary physiology in moderately preterm-born adults *JACC Cardiovasc Imaging* 2020 13 2046 2048 32417327 10.1016/j.jcmg.2020.03.016 PMC7477490

[10] Gitt AK Wasserman K Kilkowski C Kleemann T Kilkowski A Bangert M *et al.* Exercise anaerobic threshold and ventilatory efficiency identify heart failure patients for high risk of early death *Circulation* 2002 106 3079 3084 12473555 10.1161/01.cir.0000041428.99427.06

[11] Myers J Prakash M Froelicher V Do D Partington S Atwood JE Exercise capacity and mortality among men referred for exercise testing *N Engl J Med* 2002 346 793 801 11893790 10.1056/NEJMoa011858

[12] Edwards MO Kotecha SJ Lowe J Watkins WJ Henderson AJ Kotecha S Effect of preterm birth on exercise capacity: a systematic review and meta-analysis *Pediatr Pulmonol* 2015 50 293 301 29889363 10.1002/ppul.23117

[13] Haraldsdottir K Watson AM Beshish AG Pegelow DF Palta M Tetri LH *et al.* Heart rate recovery after maximal exercise is impaired in healthy young adults born preterm *Eur J Appl Physiol* 2019 119 857 866 30635708 10.1007/s00421-019-04075-z PMC7100254

[14] Vrijlandt EJLE Gerritsen J Boezen HM Grevink RG Duiverman EJ Lung function and exercise capacity in young adults born prematurely *Am J Respir Crit Care Med* 2006 173 890 896 16456146 10.1164/rccm.200507-1140OC

[15] Haraldsdottir K Watson AM Pegelow DF Palta M Tetri LH Levin T *et al.* Blunted cardiac output response to exercise in adolescents born preterm *Eur J Appl Physiol* 2020 120 2547 2554 32862247 10.1007/s00421-020-04480-9 PMC12908129

[16] Pianosi PT Fisk M Cardiopulmonary exercise performance in prematurely born children *Pediatr Res* 2000 47 653 658 10813592 10.1203/00006450-200005000-00016

[17] Clemm H Røksund O Thorsen E Eide GE Markestad T Halvorsen T Aerobic capacity and exercise performance in young people born extremely preterm *Pediatrics* 2012 129 e97 e105 22201154 10.1542/peds.2011-0326

[18] Clemm HH Vollsæter M Røksund OD Eide GE Markestad T Halvorsen T Exercise capacity after extremely preterm birth. Development from adolescence to adulthood *Ann Am Thorac Soc* 2014 11 537 545 24502400 10.1513/AnnalsATS.201309-311OC

[19] Prenzel F Vogel M Siekmeyer W Körner A Kiess W Vom Hove M Exercise capacity in children with bronchopulmonary dysplasia at school age *Respir Med* 2020 171 106102 32823240 10.1016/j.rmed.2020.106102

[20] Fick A 1870 https://digitalesammlungen.uni-weimar.de/viewer/metadata/lit26189/1/

[21] Duke JW Lovering AT Respiratory and cardiopulmonary limitations to aerobic exercise capacity in adults born preterm *J Appl Physiol (1985)* 2020 129 718 724 32790592 10.1152/japplphysiol.00419.2020 PMC7654697

[22] Huckstep OJ Williamson W Telles F Burchert H Bertagnolli M Herdman C *et al.* Physiological stress elicits impaired left ventricular function in preterm-born adults *J Am Coll Cardiol* 2018 71 1347 1356 29566820 10.1016/j.jacc.2018.01.046 PMC5864965

[23] Huckstep OJ Burchert H Williamson W Telles F Tan CMJ Bertagnolli M *et al.* Impaired myocardial reserve underlies reduced exercise capacity and heart rate recovery in preterm-born young adults *Eur Heart J Cardiovasc Imaging* 2021 22 572 580 32301979 10.1093/ehjci/jeaa060 PMC8081423

[24] Duke JW Zidron AM Gladstone IM Lovering AT Alleviating mechanical constraints to ventilation with heliox improves exercise endurance in adult survivors of very preterm birth *Thorax* 2019 74 302 304 30217953 10.1136/thoraxjnl-2018-212346

[25] Yang J Epton MJ Harris SL Horwood J Kingsford RA Troughton R *et al.* Reduced exercise capacity in adults born at very low birth weight: a population-based cohort study *Am J Respir Crit Care Med* 2022 205 88 98 34499592 10.1164/rccm.202103-0755OC

[26] Shah RV Murthy VL Colangelo LA Reis J Venkatesh BA Sharma R *et al.* Association of fitness in young adulthood with survival and cardiovascular risk: the Coronary Artery Risk Development in Young Adults (CARDIA) study *JAMA Intern Med* 2016 176 87 95 26618471 10.1001/jamainternmed.2015.6309 PMC5292201

[27] Morales Mestre N Papaleo A Morales Hidalgo V Caty G Reychler G Physical activity program improves functional exercise capacity and flexibility in extremely preterm children with bronchopulmonary dysplasia aged 4–6 years: a randomized controlled trial *Arch Bronconeumol (Engl Ed)* 2018 54 607 613 30518495 10.1016/j.arbres.2018.05.001

[28] Williamson W Huckstep OJ Frangou E Mohamed A Tan C Alsharqi M *et al.* Trial of Exercise to Prevent HypeRtension in young Adults (TEPHRA) a randomized controlled trial: study protocol *BMC Cardiovasc Disord* 2018 18 208 30400774 10.1186/s12872-018-0944-8 PMC6220491

[29] Williamson W Lewandowski AJ Huckstep OJ Lapidaire W Ooms A Tan C *et al.* Effect of moderate to high intensity aerobic exercise on blood pressure in young adults: the TEPHRA open, two-arm, parallel superiority randomized clinical trial *EClinicalMedicine* 2022 48 101445 35706495 10.1016/j.eclinm.2022.101445 PMC9112102

[30] Bush A Cramer D Guidelines for the measurement of respiratory function *Respir Med* 1994 88 798 7846347 10.1016/s0954-6111(05)80210-0

[31] Stringer WW Hansen JE Wasserman K Cardiac output estimated noninvasively from oxygen uptake during exercise *J Appl Physiol (1985)* 1997 82 908 912 9074981 10.1152/jappl.1997.82.3.908

[32] Meyer T Lucía A Earnest CP Kindermann W A conceptual framework for performance diagnosis and training prescription from submaximal gas exchange parameters—theory and application *Int J Sports Med* 2005 26 S38 S48 15702455 10.1055/s-2004-830514

[33] Riley MS Pórszász J Engelen MPKJ Brundage BH Wasserman K Gas exchange responses to continuous incremental cycle ergometry exercise in primary pulmonary hypertension in humans *Eur J Appl Physiol* 2000 83 63 70 11072775 10.1007/s004210000240

[34] Tikanmäki M Tammelin T Sipola-Leppänen M Kaseva N Matinolli HM Miettola S *et al.* Physical fitness in young adults born preterm *Pediatrics* 2016 137 20151289 10.1542/peds.2015-1289 26715606

[35] Wenchich L Zeman J Hansíková H Plavka R Sperl W Houstek J Mitochondrial energy metabolism in very premature neonates *Biol Neonate* 2002 81 229 235 12011566 10.1159/000056753

[36] Honzik T Wenchich L Böhm M Hansikova H Pejznochova M Zapadlo M *et al.* Activities of respiratory chain complexes and pyruvate dehydrogenase in isolated muscle mitochondria in premature neonates *Early Hum Dev* 2008 84 269 276 17698302 10.1016/j.earlhumdev.2006.07.008

[37] Tetri LH Diffee GM Barton GP Braun RK Yoder HE Haraldsdottir K *et al.* Sex-specific skeletal muscle fatigability and decreased mitochondrial oxidative capacity in adult rats exposed to postnatal hyperoxia *Front Physiol* 2018 9 326 29651255 10.3389/fphys.2018.00326 PMC5884929

[38] Deprez A Orfi Z Radu A He Y Ravizzoni Dartora D Dort J *et al.* Transient neonatal exposure to hyperoxia, an experimental model of preterm birth, leads to skeletal muscle atrophy and fiber type switching *Clin Sci (Lond)* 2021 135 2589 2605 34750633 10.1042/CS20210894

[39] Telles F McNamara N Nanayakkara S Doyle MP Williams M Yaeger L *et al.* Changes in the preterm heart from birth to young adulthood: a meta-analysis *Pediatrics* 2020 146 e20200146 32636236 10.1542/peds.2020-0146

[40] Tucker WJ Lijauco CC Hearon CM Jr Angadi SS Nelson MD Sarma S *et al.* Mechanisms of the improvement in peak VO_2_ with exercise training in heart failure with reduced or preserved ejection fraction *Heart Lung Circ* 2018 27 9 21 28870770 10.1016/j.hlc.2017.07.002

[41] Haykowsky MJ Brubaker PH Stewart KP Morgan TM Eggebeen J Kitzman DW Effect of endurance training on the determinants of peak exercise oxygen consumption in elderly patients with stable compensated heart failure and preserved ejection fraction *J Am Coll Cardiol* 2012 60 120 128 22766338 10.1016/j.jacc.2012.02.055 PMC3429944

[42] van Tol BAF Huijsmans RJ Kroon DW Schothorst M Kwakkel G Effects of exercise training on cardiac performance, exercise capacity and quality of life in patients with heart failure: a meta-analysis *Eur J Heart Fail* 2006 8 841 850 16713337 10.1016/j.ejheart.2006.02.013

[43] Mohamed A Marciniak M Williamson W Huckstep OJ Lapidaire W McCance A *et al.* Association of systolic blood pressure elevation with disproportionate left ventricular remodeling in very preterm-born young adults: the preterm heart and elevated blood pressure *JAMA Cardiol* 2021 6 821 829 33978675 10.1001/jamacardio.2021.0961 PMC8117059

[44] Lewandowski AJ Bradlow WM Augustine D Davis EF Francis J Singhal A *et al.* Right ventricular systolic dysfunction in young adults born preterm *Circulation* 2013 128 713 720 23940387 10.1161/CIRCULATIONAHA.113.002583

[45] Jones AM Carter H The effect of endurance training on parameters of aerobic fitness *Sports Med* 2000 29 373 386 10870864 10.2165/00007256-200029060-00001

[46] Blomqvist CG Saltin B Cardiovascular adaptations to physical training *Annu Rev Physiol* 1983 45 169 189 6221687 10.1146/annurev.ph.45.030183.001125

[47] Corrado PA Barton GP Francois CJ Wieben O Goss KN Sildenafil administration improves right ventricular function on 4D flow MRI in young adults born premature *Am J Physiol Heart Circ Physiol* 2021 320 H2295 H2304 33861148 10.1152/ajpheart.00824.2020 PMC8289359

[48] Campbell MJ Julious SA Altman DG Estimating sample sizes for binary, ordered categorical, and continuous outcomes in two group comparisons *BMJ* 1995 311 1145 1148 7580713 10.1136/bmj.311.7013.1145 PMC2551061

[49] Rothwell PM Treating individuals 2. Subgroup analysis in randomised controlled trials: importance, indications, and interpretation *Lancet* 2005 365 176 186 15639301 10.1016/S0140-6736(05)17709-5

[50] Williamson W Foster C Reid H Kelly P Lewandowski AJ Boardman H *et al.* Will exercise advice be sufficient for treatment of young adults with prehypertension and hypertension? A systematic review and meta-analysis *Hypertension* 2016 68 78 87 27217408 10.1161/HYPERTENSIONAHA.116.07431

